# Refining the CHA_2_DS_2_VASc risk stratification scheme: shall we drop the sex category criterion?

**DOI:** 10.1093/europace/euae280

**Published:** 2024-11-10

**Authors:** Hiroyuki Yoshimura, Rui Providencia, Chris Finan, Amand Floriaan Schmidt, Gregory Y H Lip

**Affiliations:** Institute of Health Informatics Research, University College London, 222 Euston Road, London NW1 2DA, UK; Institute of Health Informatics Research, University College London, 222 Euston Road, London NW1 2DA, UK; Barts Heart Centre, Barts Health NHS Trust, London, UK; Institute of Cardiovascular Science, University College London, London, UK; Institute of Cardiovascular Science, University College London, London, UK; Division Heart and Lungs, Department of Cardiology, University Medical Center Utrecht, Utrecht, The Netherlands; Liverpool Centre for Cardiovascular Science at University of Liverpool, Liverpool John Moores University and Liverpool Heart and Chest Hospital, Liverpool, UK; Department of Clinical Medicine, Danish Center for Health Services Research, Aalborg University, Aalborg, Denmark

**Keywords:** Atrial fibrillation, Thromboembolic risk prediction, Thromboembolic risk stratification score, Electronic health records

## Abstract

**Aims:**

The CHA_2_DS_2_VASc score is recommended for stroke risk stratification in patients with atrial fibrillation (AF). This score assigns one extra point to female sex based on evidence from the early 2000s, suggesting higher thromboembolic risk in women. This incremental risk of thromboembolism in women has decreased over time between 2007 and 2018, becoming non-significant in recent years. The objective of this study was to assess the impact of removing the sex category (Sc) from the CHA_2_DS_2_VASc score, thus validating a non-sex CHA_2_DS_2_VASc (i.e. CHA_2_DS_2_VA) score.

**Methods and results:**

We analysed UK primary and secondary care data comprising 195 719 patients with AF followed between 1998 and 2016 (mean age: 75.9 ± 12.3 years; 49.2% women). Among 126 428 non-anticoagulated patients, we compared the CHA_2_DS_2_VASc vs. CHA_2_DS_2_VA scores every calendar year. Throughout 413 007 patient-years, a total of 8742 events of ischaemic stroke or systemic embolism were recorded. Sex differences in thromboembolic risk were not observed in the lower-risk population, but higher stroke rates were consistently seen in female patients in the higher-risk category (i.e. CHA_2_DS_2_VA ≥2). *C*-statistics for both CHA_2_DS_2_VA and CHA_2_DS_2_VASc scores were similar over the years (ranging from 0.62 to 0.71). With CHA_2_DS_2_VA, no relevant differences were observed in integrated discrimination improvement, and net reclassification improvement (NRI) resulted in improved reclassification (11%) in lower thromboembolic risk groups. The NRI suggested misclassification in higher thromboembolic risk patients (−7%), but this did not affect their indication for anticoagulation (i.e. patients retained their high-risk status).

**Conclusion:**

Removing Sc from the CHA_2_DS_2_VASc score does not affect its ability to discriminate thromboembolic events in the population with AF. The use of CHA_2_DS_2_VA may simplify initial decision-making for thromboprophylaxis.

## Graphical Abstract

**Graphical Abstract:**
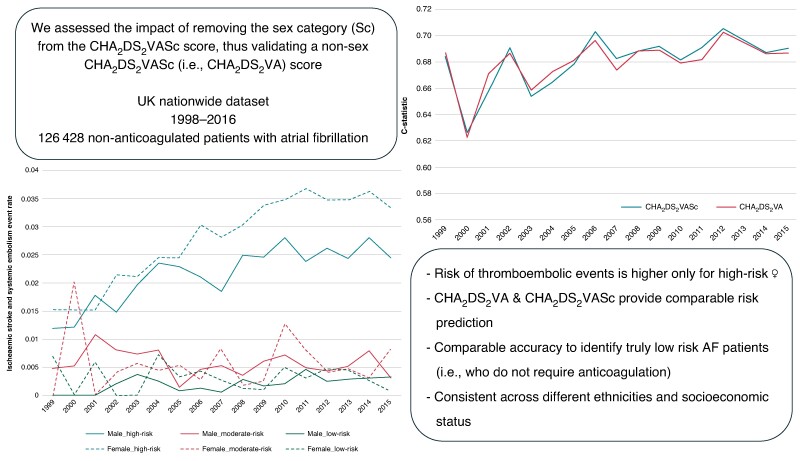
The study assesses the impact of removing the sex category from the CHA_2_DS_2_VASc score (CHA_2_DS_2_VA) for stroke risk in patients with atrial fibrillation (AF). Sex differences in thromboembolic risk were not observed in the lower-risk population with AF, but higher stroke rates were consistently seen in female patients in the higher-risk category. Removing female sex from the CHA_2_DS_2_VASc score does not compromise its discrimination performance for thromboembolic events. Accuracy of the CHA_2_DS_2_VA score to identify truly low-risk patients with AF was comparable to that of the CHA_2_DS_2_VASc score. The study suggests that using CHA_2_DS_2_VA may simplify initial decision-making for thromboprophylaxis in the population with AF.

**What’s new?**

- The Nationwide UK data show that stroke and thromboembolic risk prediction using CHA_2_DS_2_VA and CHA_2_DS_2_VASc scores is comparable.
- Accuracy of the CHA_2_DS_2_VA score to identify truly low-risk patients with AF who do not require anticoagulation is comparable to that of the CHA_2_DS_2_VASc score.
- Similarity in thromboembolic risk prediction using CHA_2_DS_2_VA and CHA_2_DS_2_VASc scores was consistent across different ethnicities and socioeconomic status.

## Introduction

Stroke and systemic embolism are among the most feared complications of atrial fibrillation (AF), and oral anticoagulation (OAC) therapy is recommended in guidelines globally for patients considered to be at moderate-to-high risk of this complication.^1–4^

The CHA_2_DS_2_VASc score is one of the most widely utilized stroke risk stratification schemes to identify such patients, including the more common and validated stroke risk factors: congestive heart failure, hypertension, age, diabetes mellitus, stroke, vascular disease, and sex category (Sc) (i.e. women are classified as higher risk).^5^ Older studies reported that women with AF were at higher cardioembolic risk than men,^6–8^ hence the incorporation of female sex into the CHA_2_DS_2_VASc score (with the Sc criterion).^9^

The original CHA_2_DS_2_VASc score publication utilized a cohort of patients from the Euro Heart Survey on AF from two decades ago, whereby patients were enrolled between 2003 and 2004.^5^ At the time, some criticism was raised regarding the small sample size of the cohort and potential lack of power to prove an association of novel risk factors when added to the CHADS_2_ risk score. A subsequent Swedish nationwide study suggested that women with AF had a higher risk of stroke compared with men, but the association was complex due to an age dependency to this risk, as women aged <65 years without any other risk factors were not at higher risk of events.^6^

Over the years, CHA_2_DS_2_VASc has been widely used and incorporated in guidelines.^1–4^ Annex A (see Supplementary material) provides a detailed description of the usage of the CHA_2_DS_2_VASc score and role of the Sc for thromboembolic risk stratification in the guidelines.

More recently, a time-trend analysis of a Finnish cohort suggested that the incremental risk of stroke observed in women has diminished between 2007 and 2018, such that a 20–30% higher stroke rate was observed for women compared with men during the first few years, and this difference was gradually attenuated and became non-significant in more recent years.^10^ It was suggested this was probably linked to other concomitant health trends, such as improvements in risk factor management and lifestyle-related factors, reflecting a more integrated approach to patients with AF, which has materialized into the AF better care pathway: avoid stroke, better symptom management, and cardiovascular and comorbidity risk reduction.^11^

The recently published European Society of Cardiology guidelines recommended the utilization of a non-sex CHA_2_DS_2_VASc score (i.e. CHA_2_DS_2_VA) based on consensus opinions of the experts (level of evidence C).^12^ Using linked UK electronic health records over a two-decade period, we aimed to assess whether dropping the Sc from the CHA_2_DS_2_VASc risk score affects its discrimination and performance.

## Methods

### Data sources

We analysed a linked nationwide electronic health record data set from the UK, including: primary care records from the Clinical Practice Research Datalink (CPRD), secondary care records from the Hospital Episodes Statistics (HES), and death census from the Office for National Statistic.^13^ To identify comorbidities, the Health Data Research UK Phenotype Library,^14^ which defines phenotypes based on we utilized READ and/or SNOMED CT codes, and International Classification of Diseases Tenth Revision (ICD-10) code.

### Study population

Participants were enrolled from 1 January 1998 to 31 May 2016. After excluding participants in CPRD who were not linked to HES and were under 18 years old, the population consisted of 6 529 382 individuals. There were 195 719 patients with new-onset AF diagnosis in primary and secondary care during the study period.

Thromboembolic risk at the first assessment has potential for change due to developing comorbidities over the years.^15^ Therefore, to address potential biases arising from temporal or dynamic changes in stroke risk, we segmented our study periods into annual short observation periods, with the CHA_2_DS_2_VASc and CHA_2_DS_2_VA scores being calculated on January 1 in every year. This is in line with global guideline recommendations for assessing thromboembolic risk regularly.^1–4^

Participants not on OAC therapy were eligible for the study and were followed from January 1 until the earliest date of the following: death, transfer out clinical practice, OAC initiation, stroke occurrence, or study end (December 31). All available follow-up for each patient was considered. To clarify, 1 year of follow-up was assigned to each calendar year, and patients 5 years of follow-up (to exemplify), starting in 2001, had follow-up in 2001, 2002, 2003, 2004, and 2005.

We excluded participants who initiated OAC therapy within the initial 3-month period from the analysis to mitigate censoring bias. This step was necessary due to potential issues with prescription data during the first 3 months of the year: calendar year reset with clustering of prescription records in January and subsequent months, data entry delays, annual review and changes of primary practice prescription policies, and administrative issues leading to prescription coverage gaps. These factors could contribute to patients on OACs being wrongly classified as not on OAC for a short period of time and subsequently censored on the date of their first prescription for that given year. As this may occur in a fair number of patients, it could lead to an important censoring bias. As such, we elected to remove patients with OAC prescriptions in the first 3 months for a given year from the analysis.

The study’s primary outcome was a composite of ischaemic stroke and systemic embolism, which are recorded in ICD-10 code and READ codes (see Supplementary material online, *Table S1*).

To identify socioeconomic deprivation, we utilized the English indices of deprivation 2015.^16^ These measures of relative deprivation in small areas across the UK were divided into quintiles. For ethnicity, the six categories were classified into two groups: white and non-white. Non-white included Asian, Black, Mixed, and Other. As subgroup analyses, we performed two separate comparisons: white vs. non-white and the most deprived quintile vs. least deprived quintile.

A CHA_2_DS_2_VASc score of 0 for men and 1 for women was considered low risk (no indication for OAC), a score of 1 for men and 2 for women was considered moderate risk (should be considered for OAC), and a score of 2 or above for men and 3 and above for women was considered high risk (oral anticoagulant is recommended).^1,2^

A sensitivity analysis was performed including participants who initiated OAC therapy within the initial 3-month period.

### Statistical methods

Proportion (%) were utilized for categorical variables and means with standard deviation (or median, interquartile range) for continuous variables. Incidence rates were estimated by survival analysis for ischaemic stroke and systemic embolism. Multivariate Cox regression was performed to assess the annual association of women with thromboembolic risk, adjusting for age and CHA_2_DS_2_VASc score. We compared annual thromboembolic risk discrimination utilizing the two risk stratifications schemes, CHA_2_DS_2_VASc score vs. CHA_2_DS_2_VA, using *C*-statistics, net reclassification improvement (NRI), and integrated discrimination improvement (IDI). Calibration plots were traced. We merged the data sets from 1999 to 2015 into a comprehensive data set to assess the effects within specific sub-groups: stroke risk, ethnicity, and socioeconomic deprivation.

Harrell’s *C*-index was used to evaluate the risk models in survival analysis. Net reclassification improvement and IDI measure risk refinement were provided by a new marker or classification. Integrated discrimination improvement is related to the mean improvement in risk prediction, while NRI illustrates the proportion of individuals with changed prediction. Net reclassification improvement was calculated to assess reclassification offered by a new risk prediction model compared with an existing model (e.g. how patients with and without events shift from higher- to lower-risk categories, or vice versa).^17^ In this analysis, we assessed continuous NRI applying time-to-event data.^18^ Positive values indicate improved classification (i.e. patients with events having higher scores or patients without events having lower scores), and negative values indicate worsened classification (i.e. patients with events having lower scores or patients without events having higher scores). Integrated discrimination improvement is calculated as the difference between the increase in sensitivity and the decrease in false-positive rate (1-specificity) provided by a new risk prediction model compared with an existing model.^17^

All statistical analyses were performed using R 4.3.0 and RStudio 1.2. To calculate NRI and IDI, we utilized survIDINRI packages.^19^

Due to numerous comparisons being made simultaneously when assessing time trends, we applied the Bonferroni correction to adjust for multiple comparisons and adjusted the significance level of the *P*-value threshold to minimize chances of Type I error. The adjusted significance level was the *P*-value of 0.05 divided by the number of statistical tests executed—2.94 × 10^−3^ (i.e. 0.05/17 due to 17 years being assessed separately).

## Results

There were 195 719 patients with a new-onset AF diagnosis (mean age: 75.9 ± 12.3 years; 49.2% women; CHA_2_DS_2_VASc score: 3.6 ± 1.8) (*Table 1*). Over 97% of participants were white ethnicity. Women were on average 5.9 years older and had a CHA_2_DS_2_VASc score 1.3 points higher than men.

**Table 1:** Study population demographics and baseline comorbidities for all patients with AF (left) and patients with AF not on OAC (right)

	New-onset AF				Patients with AF not on OAC		
	Overall (*n* = 195 719)	Not on OAC (*n* = 126 428)	On OAC (*n* = 69 291)	*P*	Men (*n* = 60 512)	Women (*n* = 65 916_	*P*
Age	75.9 (12.3)	77.6 (12.8)	72.7 (10.6)	<0.001	74.5 (13.3)	80.4 (11.7)	<0.001
Men	99 490 (50.8%)	60 512 (47.9%)	38 978 (56.3%)	<0.001	—	—	—
White	179 393 (97.6%)	114 347 (97.4%)	65 046 (98.0%)	<0.001	54 972 (97.1%)	59 375 (97.6%)	<0.001
Asian	1931 (1.1%)	1359 (1.2%)	572 (0.9%)		751 (1.3%)	608 (1.0%)	
Black	960 (0.5%)	655 (0.6%)	305 (0.5%)		348 (0.6%)	307 (0.5%)	
Mixed	286 (0.2%)	200 (0.2%)	86 (0.1%)		113 (0.2%)	87 (0.1%)	
Other	1260 (0.7%)	867 (0.7%)	393 (0.6%)		434 (0.8%)	433 (0.7%)	
Unknown	11 889	9000	2889		3894	5106	
Most deprived quintile	31 478 (16.1%)	19 930 (15.8%)	11 548 (16.7%)	<0.001	9648 (15.9%)	10 282 (15.6%)	0.2
Least deprived quintile	45 256 (23.1%)	29 529 (23.4%)	15 727 (22.7%)		14 167 (23.4%)	15 362 (23.3%)	
Heart failure	43 236 (22.1%)	30 370 (24.0%)	12 866 (18.6%)	<0.001	13 946 (23.0%)	16 424 (24.9%)	<0.001
Hypertension	122 589 (62.6%)	79 875 (63.2%)	42 714 (61.6%)	<0.001	35 950 (59.4%)	43 925 (66.6%)	<0.001
Diabetes	31 795 (16.2%)	21 589 (17.1%)	10 206 (14.7%)	<0.001	11 501 (19.0%)	10 088 (15.3%)	<0.001
Stroke	47 142 (24.1%)	31 093 (24.6%)	16 049 (23.2%)	<0.001	13 966 (23.1%)	17 127 (26.0%)	<0.001
Vascular disease	38 807 (19.8%)	26 557 (21.0%)	12 250 (17.7%)	<0.001	15 307 (25.3%)	11 250 (17.1%)	<0.001
Antiplatelet use	103 949 (53.1%)	67 066 (53.0%)	36 883 (53.2%)	0.4	32 877 (54.3%)	34 189 (51.9%)	<0.001
CHA_2_DS_2_VASc (mean)	3.6 (1.8)	3.8 (1.8)	3.3 (1.8)	<0.001	3.1 (1.8)	4.4 (1.6)	<0.001
CHA_2_DS_2_VASc				<0.001			<0.001
0	9241 (4.7%)	5440 (4.3%)	3801 (5.5%)		5440 (9.0%)	0 (0.0%)	
1	16 786 (8.6%)	9261 (7.3%)	7525 (10.9%)		6214 (10.3%)	3047 (4.6%)	
2	27 225 (13.9%)	15 257 (12.1%)	11 968 (17.3%)		11 302 (18.7%)	3955 (6.0%)	
3	39 394 (20.1%)	24 610 (19.5%)	14 784 (21.3%)		13 407 (22.2%)	11 203 (17.0%)	
4	42 348 (21.6%)	28 289 (22.4%)	14 059 (20.3%)		10 328 (17.1%)	17 961 (27.2%)	
5	29 056 (14.8%)	20 225 (16.0%)	8831 (12.7%)		7788 (12.9%)	12 437 (18.9%)	
6	19 782 (10.1%)	14 303 (11.3%)	5479 (7.9%)		4072 (6.7%)	10 231 (15.5%)	
7	8791 (4.5%)	6590 (5.2%)	2201 (3.2%)		1574 (2.6%)	5016 (7.6%)	
8	2692 (1.4%)	2123 (1.7%)	569 (0.8%)		387 (0.6%)	1736 (2.6%)	
9	404 (0.2%)	330 (0.3%)	74 (0.1%)		0 (0.0%)	330 (0.5%)	

**Figure 1:**
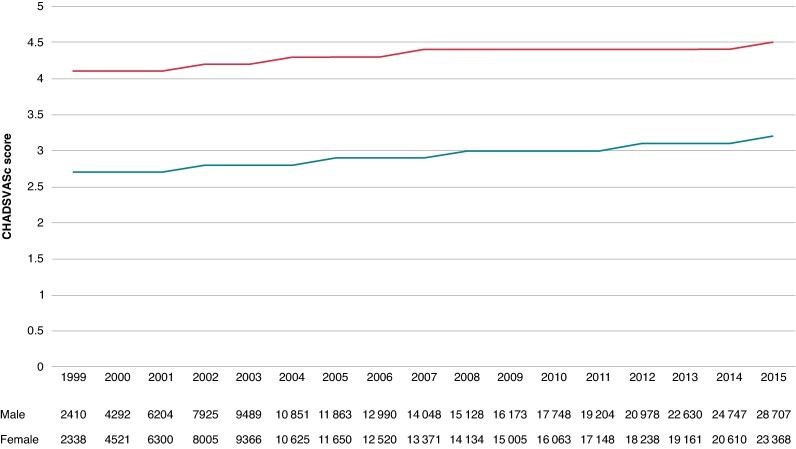
Time trends in CHA_2_DS_2_VASc score and number of patients being follow-up during the study period. The bar graphs represent the number of participants by sex enrolled in this study on January 1 of each year. More participants were present in the second half of the study. The line plot represents the average CHA₂DS₂VASc scores for the enrolled participants, assessed on January 1 of each year. CHA_2_DS_2_VASc score was ∼1 point higher for women, and the score increased for both men and women by ∼ 0.3 throughout the study period.

The prevalence of heart failure, hypertension, and stroke was higher in women, and diabetes, vascular disease, and antiplatelet use were more frequent in men. *Figure 1* depicts an upward trend in both the number of participants included in the analysis and their mean CHA_2_DS_2_VASc score across the years.

Anticoagulants were used by 69 291 patients (35.4%). Anticoagulant recipients were more frequently men, 5 years younger in average, and had a lower CHA_2_DS_2_VASc score than non-anticoagulated patients (3.3 ± 1.8 vs. 3.8 ± 1.8; *P* < 0.001) (*Table 1* and Supplementary material online, *Table S2*).

### Female vs. male differences in stroke rates

Among 126 428 non-anticoagulated patients, there were a total of 16 326 thromboembolic events, with 8742 individuals experiencing at least one thromboembolic event over a period of 413 007 patient-years (7747 ischaemic strokes and 995 systemic embolism events). Annual event rates per 100 patient-years, cumulative incidence rates, and without censoring are presented in Supplementary material online, *Table S3*.

**Figure 2:**
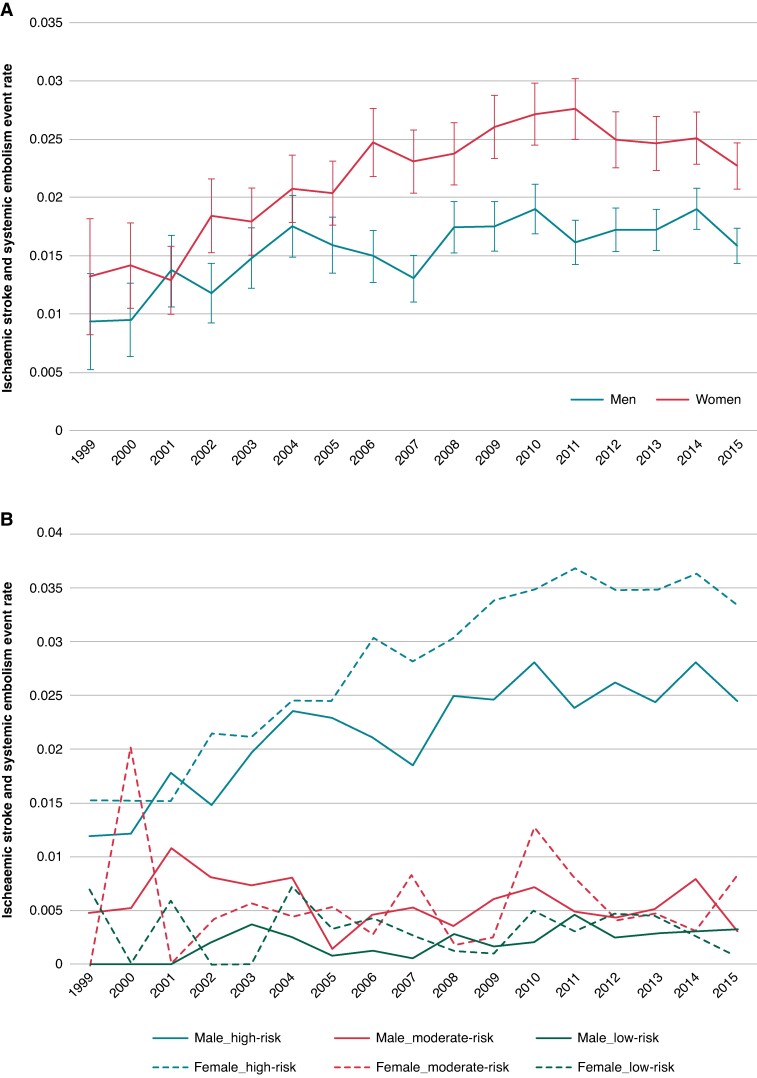
(*A*) Comparison of stroke and systemic embolism rate men vs. women over study period. (*B*) Comparison of stroke and systemic embolism stratified by assigned risk category using CHA_2_DS_2_VASc score. *A* shows that female sex (all CHA₂DS₂VASc scores) has an increased event rate throughout the study period. *B* stratifies patients by risk category showing that the increase in risk is driven by patients in the high-risk category: lines for men and women overlap for low and intermediate risk, and event rate is higher for women with higher risk when compared with men.

When comparing the overall annual ischaemic stroke and systemic embolism event rates, women had higher event rates every year, except for 2001, with differences ranging between 0.3 and 1.1% (*Figure 2A*). After adjustment for age and CHA_2_DS_2_VASc, the annual excess of events in women became less pronounced failing to reach the significance threshold in some years (see Supplementary material online, *Table S4*).

The annual ischaemic stroke and systemic embolism events rate stratified by sex and risk category using CHA_2_DS_2_VASc score showed higher event rates among women vs. men in the high-risk group (CHA_2_DS_2_VASc score ≥2 for men and ≥ 3 for women, categories with indication for anticoagulants) overall. The female vs. male event rates were comparable and non-significantly different for the low- and moderate-risk groups (*Figure 2B*).

### Comparisons of CHA_2_DS_2_VASc and CHA_2_DS_2_VA

**Figure 3:**
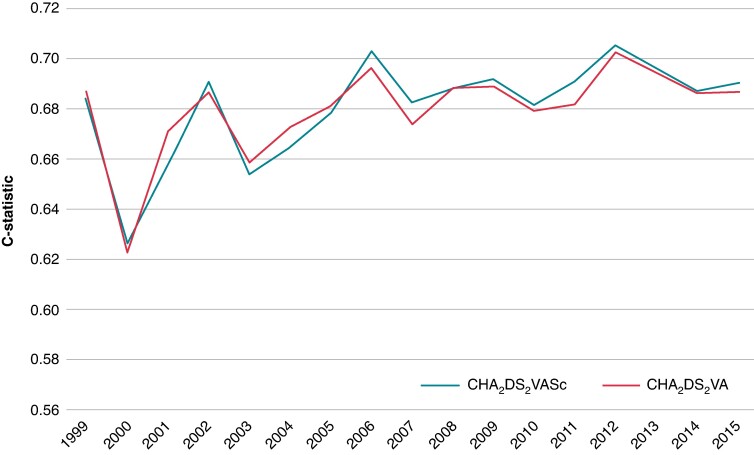
Time trends in discrimination (*C*-statistics) for both risk stratification schemes.

Annual *C*-index values were comparable for both scores, ranging between 0.62 and 0.71 (*Figure 3*). No differences in NRI were observed in the first 6 years (except in 2002), but afterwards NRI showed 6–15% less effective reclassification using the CHA_2_DS_2_VA risk scheme (*Table 2*). Integrated discrimination improvement was slightly lower (−0.001, *P* < 0.05) in 2007 and 2011, while there were no significant differences in IDI in other follow-up years. The calibration plots for CHA_2_DS_2_VA and CHA_2_DS_2_VASc scores showed good alignment between predicted and observed outcomes (see Supplementary material online, *Figure S1*).

**Table 2:** Reclassification CHA_2_DS_2_VA vs. CHA_2_DS_2_VASc measured through NRI and IDI during the study period

	IDI	95% CI	*P*-value	Continuous NRI	95% CI	*P*-value^a^
1999	0	−0.002, 0.002	0.751	−0.081	−0.213, 0.157	0.312
2000	0	−0.001, 0.001	0.505	−0.1	−0.197, 0.061	0.12
2001	0.001	0, 0.002	0.066	0.026	−0.066, 0.234	0.651
2002	−0.001	−0.002, 0	0.219	−0.11	−0.178, −0.043	0.007
2003	0	−0.001, 0.001	0.744	−0.05	−0.104, 0.065	0.1
2004	0	−0.001, 0.001	0.631	−0.04	−0.1, 0.077	0.146
2005	0	−0.001, 0.001	0.684	−0.062	−0.112, −0.014	0.013
2006	−0.001	−0.002, 0	0.073	−0.126	−0.17, −0.074	< 0.001
2007	−0.001	−0.002, 0	< 0.001	−0.144	−0.19, −0.093	<0.001
2008	0	−0.001, 0.001	0.831	−0.081	−0.12, −0.038	<0.001
2009	−0.001	−0.002, 0	0.073	−0.104	−0.144, −0.062	<0.001
2010	0	−0.001, 0	0.239	−0.091	−0.128, −0.053	<0.001
2011	−0.001	−0.002, −0.001	0.007	−0.137	−0.171, −0.105	<0.001
2012	−0.001	−0.002, 0	0.159	−0.097	−0.138, −0.066	<0.001
2013	−0.001	−0.001, 0	0.14	−0.093	−0.127, −0.054	<0.001
2014	0	−0.001, 0.001	0.877	−0.076	−0.11, −0.039	<0.001
2015	0	−0.001, 0	0.173	−0.092	−0.124, −0.057	<0.001

^a^Adjusted *P*-value for multiple comparisons: 2.94 × 10^−3^ (i.e. 0.05/17).

CI, confidence interval; IDI, integrated discrimination improvement; NRI, net reclassification improvement.

No differences in NRI were observed in the first 6 years (except in 2002), but afterwards NRI showed 6–15% less effective reclassification using the CHA_2_DS_2_VA risk scheme. This less effective reclassification means that some participants with thromboembolic events were assigned 1 point less. Integrated discrimination improvement was slightly lower (−0.001, *P* < 0.05) in 2007 and 2011, while there were no significant differences in IDI in other follow-up years. This lower IDI means that CHA_2_DS_2_VA risk scheme has lower sensitivity or higher false-positive rates compared with CHA_2_DS_2_VASc.

A sensitivity analysis including also participants who initiated OAC therapy within the initial 3-month period showed similar results to the main analysis: higher event rates in women (see Supplementary material online, *Tables S5*), and comparable performance of CHA_2_DS_2_VASc and CHA_2_DS_2_VA (see Supplementary material online, *Table S6*).

### Whole population

When comparing CHA_2_DS_2_VA vs. CHA_2_DS_2_VASc in the whole population, a lower NRI overall was observed, while IDI was unchanged (*Table 3*). Stratification of patients by thromboembolic risk categories showed that improved reclassification (positive NRI) was observed in the low- and moderate-risk group, and misclassification occurred only in the higher-risk group, but resulting in no clinical consequences as these patients still remained high risk, and their OAC indication was unchanged. The *C*-indexes were comparable for the two scores.

**Table 3:** Reclassification CHA_2_DS_2_VA vs. CHA_2_DS_2_VASc measured through NRI and IDI during the study period

	*C*-index		IDI and continuous NRI					
	CHA_2_DS_2_VASc	CHA_2_DS_2_VA	IDI	95% CI	*P*-value	Continuous NRI	95% CI	*P*-value
All population	0.69	0.69	0	−0.001, 0	0.193	−0.092	−0.102, −0.081	<0.001
Stroke risk								
High risk	0.64	0.64	0	−0.001, 0	0.551	−0.068	−0.078, −0.056	<0.001
Low and moderate risk	0.58	0.59	0	0, 0	0.266	0.111	−0.058, 0.174	0.1
Socioeconomic deprivation								
Most deprived	0.71	0.71	0	−0.001, 0	0.233	−0.099	−0.128, −0.073	<0.001
Least deprived	0.68	0.68	0	−0.001, 0	0.252	−0.068	−0.093, −0.046	<0.001
Ethnicity								
White	0.69	0.68	0	−0.001, 0	0.439	−0.092	−0.103, −0.081	<0.001
Non-white	0.71	0.72	0	−0.001, 0.002	0.97	−0.016	−0.091, 0.148	0.704

CI, confidence interval; IDI, integrated discrimination improvement; NRI, net reclassification improvement.

### Subgroup analysis

We conducted subgroup analyses to examine the predictive performance of CHA_2_DS_2_VA and CHA_2_DS_2_VASc score classification by estimated thromboembolic risk, ethnicity, and socioeconomic deprivation status. After dividing patients according to CHA_2_DS_2_VASc score values into low- to moderate-risk strata, IDI and NRI values showed a non-significant trend towards reclassification by using CHA_2_DS_2_VA into lower-risk categories among patients who did not have events and were originally classified as high risk.

The *C*-index and IDI values for white and non-white, as well as most and least deprived quintiles, were comparable (see Supplementary material online, *Figure S2*). Lower NRI values were observed in both socioeconomic deprivations groups, while non-white group (with smaller numbers) exhibited wider 95% confidence intervals.

## Discussion

Our study has four major findings, as follows: (i) the incidence of thromboembolic events in the population with low and moderate CHA_2_DS_2_VASc risk score was similar between men and women, while a tendency for higher thromboembolic events in women compared with men was observed in the population with high CHA_2_DS_2_VASc risk scores (which was more pronounced during the second half of the follow-up); (ii) overall stroke and thromboembolic risk prediction using CHA_2_DS_2_VA and CHA_2_DS_2_VASc scores was comparable; (iii) accuracy of the CHA_2_DS_2_VA score to identify truly low-risk patients with AF who do not require anticoagulation was comparable to that of the CHA_2_DS_2_VASc score; and (iv) the similarity in thromboembolic risk prediction using CHA_2_DS_2_VA and CHA_2_DS_2_VASc scores was consistent across different ethnicities and socioeconomic status.

Our study design, with year-by-year analysis of score performance, shows that the CHA_2_DS_2_VA score performed comparably to CHA_2_DS_2_VASc not only during recent years, but during most of the follow-up period. These findings are consistent with a previous study in Denmark, which suggested the CHA_2_DS_2_VA score could be used to guide decisions on initiation of OAC therapy, as the Sc component only accentuates the risk in women who would already be eligible for anticoagulation (i.e. women with CHA_2_DS_2_VA of ≥2), acting as a risk modifier rather than as a risk factor itself.^20^ Our study also showed comparable thromboembolic risk prediction between the risk stratification with or without Sc, notwithstanding that women in the higher thromboembolic risk category (and eligible for OAC) still had a higher risk of stroke than men. Furthermore, our analysis showed significant IDI differences, but these were of very small magnitude (0.001 values), and hence unlikely to carry any clinical significance.

Prior studies have investigated the rates of ischaemic stroke in females vs. males, with conflicting results.^9^ The J-RHYTHM registry in the Japanese population suggested that female sex was not associated with a higher thromboembolic risk, and removing the Sc criterion did not affect the performance of the risk stratification scheme in this study.^21,22^ However, this cohort determined anticoagulation use (or non-use) only at baseline, with no accounting for follow-up anticoagulation use, which would impact event rates. A retrospective nationwide Swedish cohort study reported that thromboembolic risk in women varies with age, with a higher risk in those aged over 75 years (i.e. with a CHA_2_DS_2_VA score of 2 or more, and hence with indication for OAC even in the absence of any risk factors besides age), and the difference was diminishing in younger age groups.^7^ A study from Taiwan’s National Health Insurance Research Database also indicated that women aged over 75 years had a higher ischaemic stroke risk than men, while women aged under 65 years had a lower ischaemic stroke risk than men.^23^

It has been previously suggested that women are less likely to receive OAC treatment than men, even when they are considered to be at higher risk of having a stroke.^24,25^ A network meta-analysis of five randomized clinical trials of patients with AF suggested a higher risk of stroke and systemic embolism and a lower risk of major bleeding in women compared with men.^26^ However, recent real-world prospective data reported no sex differences in the stroke and symptomatic thromboembolic events.^27,28^ The findings from these previous studies perhaps suggest that initiating direct oral anticoagulants based on appropriate risk stratification may help in reducing health inequity related to sex and lead to lower thromboembolic events in females.

It is unlikely that the changing relationship between female sex and stroke risk is explained by changes in female biology over recent years. Similar to what has been described for cardiac conditions, it is likely that the improvement in risk factor management (e.g. total cholesterol, systolic blood pressure, smoking) and lifestyle-related factors (e.g. physical inactivity) may play an important role.^29,30^ However, the explanation may go beyond the decrease in inequalities in ischaemic stroke risk factor management, and progress in reducing sex inequalities at a socioeconomic level may also play a role.^31^

Several reports have suggested reasons why women have a higher risk of stroke compared with men.^32,33^ One longitudinal cohort study from the USA reported a nearly two-fold stronger association between hypertension severity and incidence of ischaemic stroke in women compared with men.^34^ A community-based cohort study has also suggested that the risk of stroke and cardiovascular disease risk was increased at lower thresholds of systolic blood pressure in women compared with men.^35^ Health inequity is also a contributing factor, with studies indicating that women less commonly receive essential treatments for stroke prevention, such as OACs and statins, compared with men.^24,25,36^ Additionally, women-specific risk factors, such as both exogenous and endogenous oestrogen exposure, may explain the stroke risk differences, whereby the use of oral contraceptive pills and menopausal hormone replacement therapy (HRT) has been associated with a higher risk of ischaemic stroke.^37,38^ However, this relationship to HRT was not seen in women with AF from the Atrial Fibrillation Follow-Up Investigation of Rhythm Management (AFFIRM) trial data.^39^

Our sub-analysis showed the consistent thromboembolic risk prediction performance over time by ethnicity and socioeconomic status, suggesting that the risk prediction model using CHA_2_DS_2_VA score may be useful and effective across different ethnicities and socioeconomic groups. A retrospective analysis in the USA showed that, despite higher risk of events among African Americans, a comparable predictive ability of CHA_2_DS_2_VASc score was observed for whites, African Americans, and Hispanics.^40^ On the other hand, previous studies from east Asia suggested that CHA_2_DS_2_VASc score was useful for stroke risk stratification, but the risk might be underestimated for low-risk individuals (scores between 0 and 2).^41^ Accordingly, a modified mCHA_2_DS_2_VASc score has been proposed (with 1 point assigned to age between 50 and 74 years).^42^ These previous findings support our preliminary findings suggesting the consistency of thromboembolic risk prediction for different ethnicity and socioeconomic status in the UK population. However, further validation may be required in non-European cohorts due to the known impact of ethnicity on stroke and bleeding.^43,44^

The discriminative capacity for CHA_2_DS_2_VASc and CHA_2_DS_2_VA was low to moderate. The main advantage of CHA_2_DS_2_VASc and CHA_2_DS_2_VA is the simplicity of use and capacity to identify low-risk patients. However, the underlying oversimplification may contribute to the failure to capture the full complexity of thromboembolic risk stratification. Adjusting to the region of the globe or ethnicity, being dynamic, adding social determinants, and incorporating aspects from proteomics, genomics, and imaging might be ways of improving these scores that should be explored in the future.

### Limitations

There are several limitations to consider in this analysis. First, since the data were collected from 1999 to 2016, the rate of thromboembolic events might have changed in more recent years due to the improvements in patient management. Also, low utilization of anticoagulants in patients with AF reflects the UK reality during that period. Over the past 25 years, landmark publications and events have occurred^45^ that led to an increase in anticoagulant prescription: the publication of the BAFTA trial and CHA_2_DS_2_VASc dates from 2007 and 2011,^5,46^ and the approval of the direct oral anticoagulants for the treatment of AF, which occurred between 2012 and 2015 in the UK. Sudlow *et al*.^47^ in the late 1990s reported that only 18% of patients with AF in a UK survey were being treated with warfarin, and anticoagulant use was more frequent among patients aged 65–74 years (44%) than for those aged 75 years or above (11%). Wu *et al.*^48^ have used the CPRD data set to assess time trends in anticoagulant use among patients with AF in the UK between 2009 and 2018 and showed that the prescription rate rose from 47% in 2009 to 75% in 2018. An annual increase was observed mainly after 2013 (+5% per year compared with +1 to 2% in the initial years), coinciding with the availability of the direct oral anticoagulants. Secondly, thromboembolic risk scores can change due to ageing and the development of incident comorbidities during the observation period. We tried to adjust for this by performing annual analyses, with updated annual risk scores, to mitigate this. Thirdly, the evaluation of risk prediction models from measures such as the *C*-statistics and NRI may be biased at times.^49,50^ In this analysis, repeated assessment of risk prediction schemes over time showed the same trends and results, thereby increasing the robustness of our findings. Fourthly, the CPRD data set was collected in the UK, with over 97% of participants being white, raising the question of whether these findings may not generalize to other ethnic groups; the very low rate of non-white individuals (3%), contrasts to the 18% in the 2021 UK Census, and raises the issue of the recording of ethnicity data in the CPRD data set. A recent report by the Race Equality Foundation and the Office of National Statistics identified contributing reasons (e.g. patients refusing or preferring not to report ethnicity, fear of racial discrimination, variation in understanding of ethnicity, categories ‘unspecified’ or ‘unknown’ recorded on patients’ records, etc.) and provided recommendations and directions provided to improve the recording of ethnicity in healthcare and in future CPRD data sets.^51^ Despite this, the data in our data set allow us to provide an exploratory analysis of the topic in non-white ethnicity, corroborated by previous studies reporting that CHA_2_DS_2_VASc score is effective for stroke risk prediction in different ethnicities.^22,40,41^ Finally, the CPRD data set does not capture the use of over-the-counter medication such as aspirin. However, in the UK, patients tend to use paracetamol and ibuprofen most often as painkillers. In the absence of official UK data, an Irish study has estimated that aspirin may account for <5% of the choices among patients that take over-the-counter painkillers.^52^

## Conclusions

Removing female sex from the CHA_2_DS_2_VASc score does not compromise its discrimination performance for thromboembolic events. The use of CHA_2_DS_2_VA, with a sexless stratification of patients into three categories (0 = low risk, 1 = moderate risk, and ≥2 = high risk), may simplify initial decision-making for thromboprophylaxis.

## Supplementary Material

euae280_Supplementary_Data

## Data Availability

Data for this study were provided by United Kingdom’s Medicines and Healthcare Products Regulatory Agency following approval by the Independent Scientific Advisory Committee [17_205] and can be made available to other researchers following application via the CPRD website (https://www.cprd.com/).
