# Supplementary material for: Refining the CHA2DS2VASc risk stratification scheme: shall we drop the sex category criterion?
Source: Europace. 2024 Nov 10;26(11):euae280. doi: 10.1093/europace/euae280 (PMC11574618; doi:10.1093/europace/euae280)
Supplement: euae280_Supplementary_Data [file euae280_supplementary_data.docx]

**Supplementary Material**

**
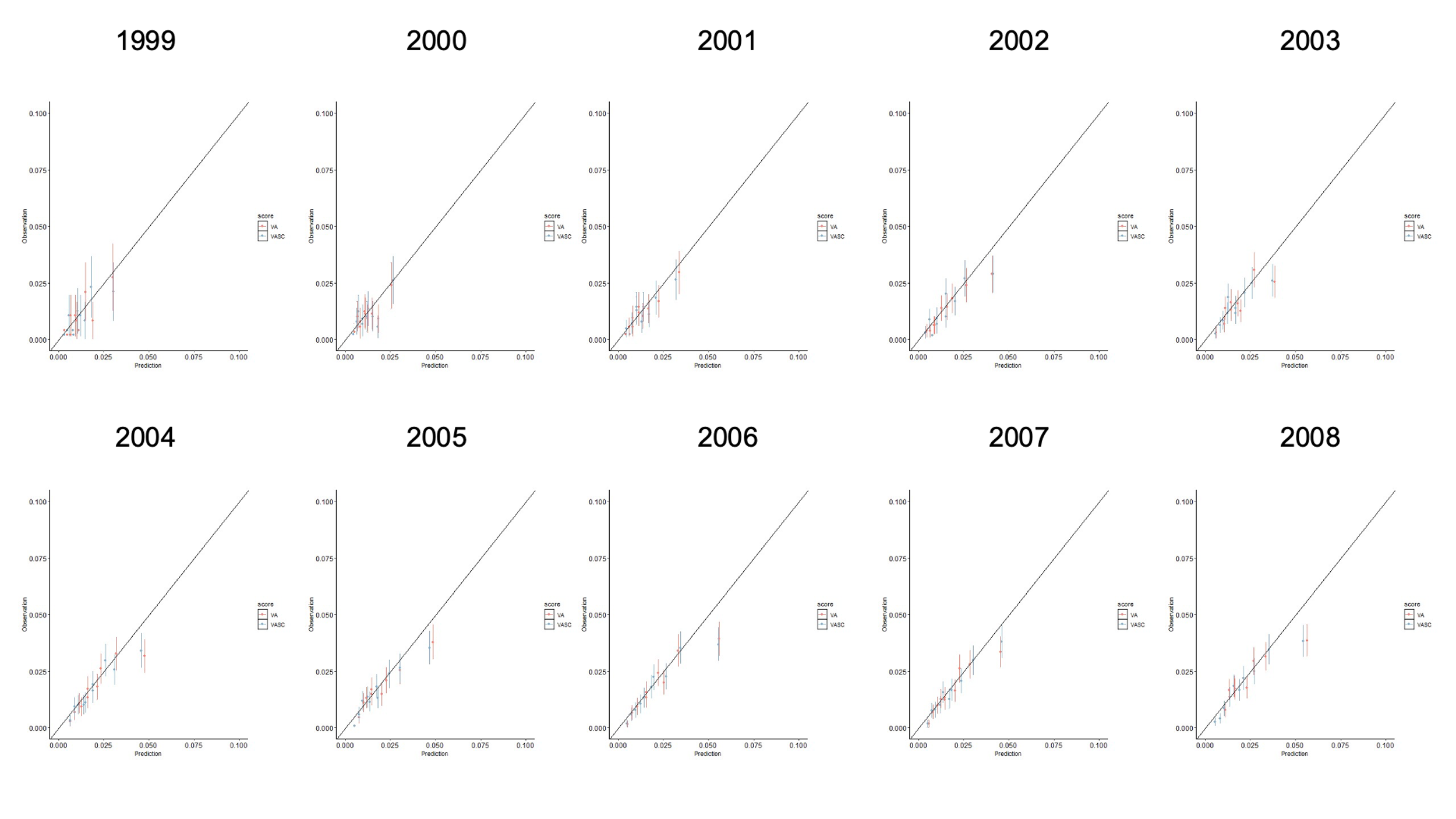
Supplementary Figure 1 –** Annual calibration plots for CHA_2_DS_2_VASc and CHA_2_D_2_VAS score from 1999 to 2015


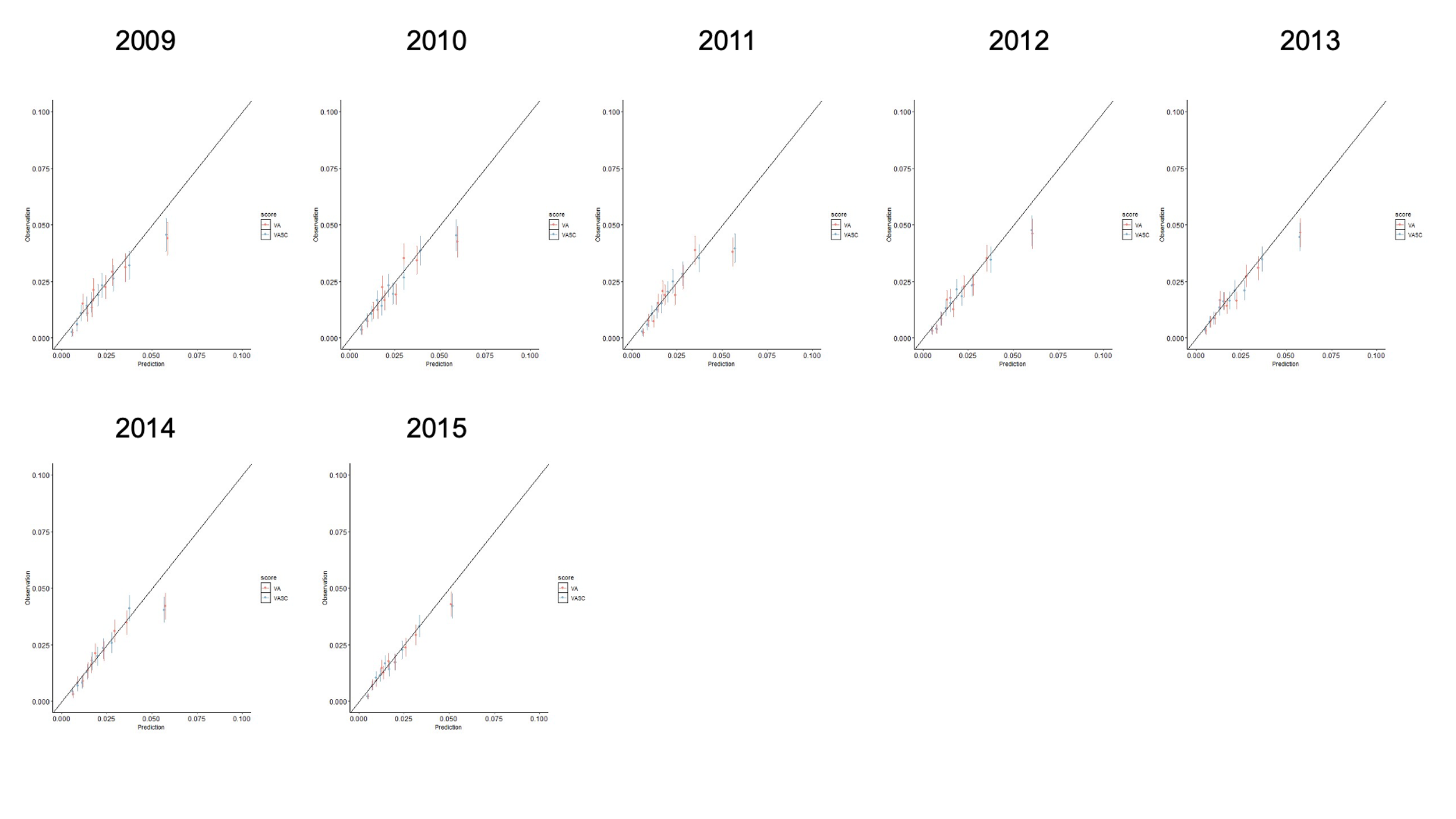


**Supplementary Figure 2 –** Time trends in discrimination (c-statistic) for both risk stratification schemes by ethnicity and socioeconomic deprivation.

A. White, B. Non-White, C. Most deprived quintile, D. Least deprived quintile

**
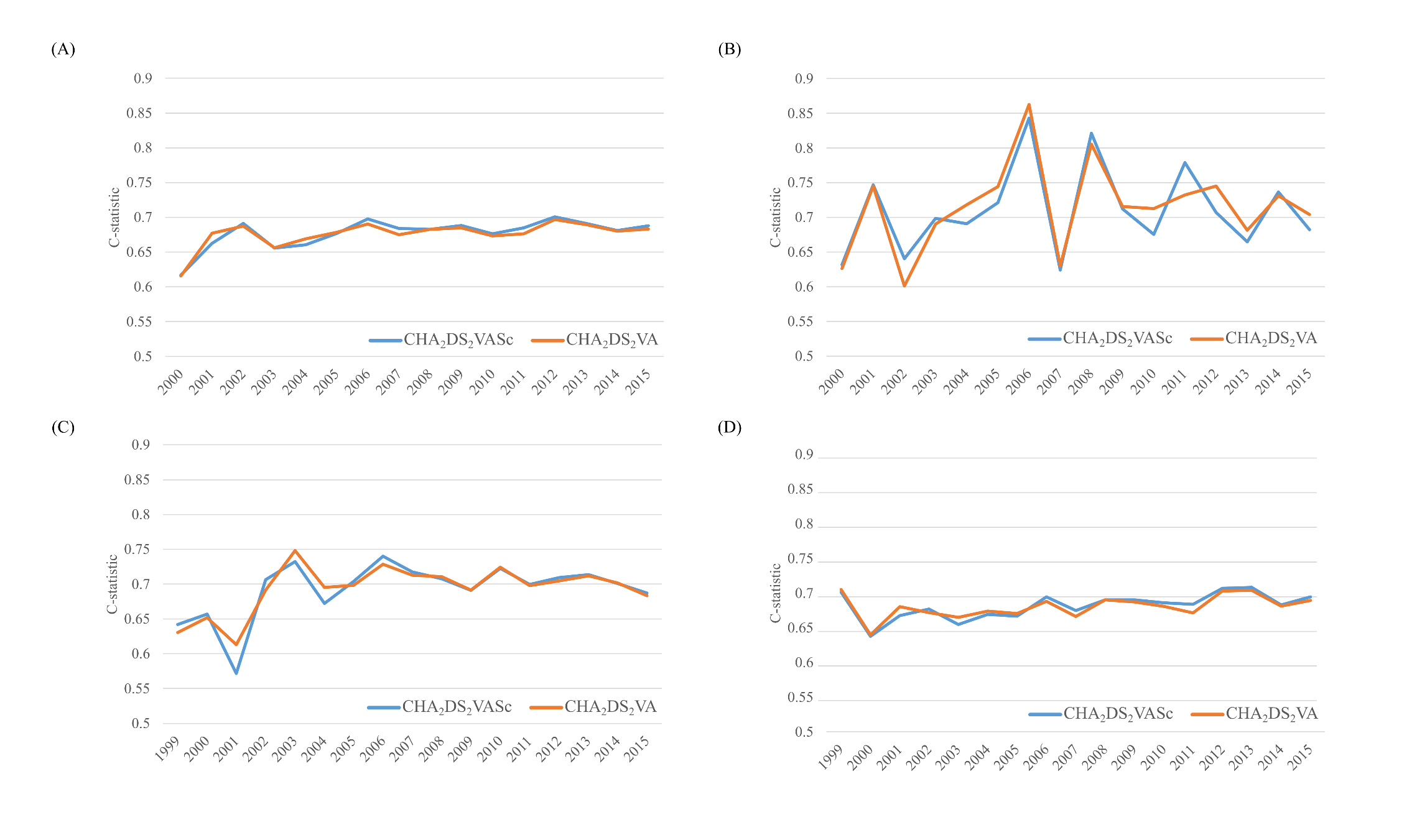
**

**Supplementary Table 1 –** List of READ and ICD-10 codes of interest

|  | **CPRD**  **READ codes** | **HES**  **ICD-10 codes** |
| --- | --- | --- |
| **Atrial Fibrillation** | G573200, G573400, G573500, 3272.00, G573000, G573300, G573.00, G573z00, 3273.00, G573100 | I48 |
| **Ischemic stroke** | G683.00, G64..11, G64..13, G671.00, G6W..00, G6X..00, Gyu6300, Gyu6400, Gyu6500, Gyu6600, Gyu6G00 | I69.3, I63 |
| **Systemic embolism** | - | I74 |

**Supplementary Table 2 –** Demographics and baseline comorbidities in patients treated with OAC

|  | Overall  (n=69,291) | Men  (n=38,978) | Women  (n=30,313) | P |
| --- | --- | --- | --- | --- |
|  |  |  |  |  |
| Age | 72.7 (10.6) | 70.8 (10.8) | 75.1 (9.8) | <0.001 |
| Ethnicity |  |  |  | 0.2 |
| White | 65,046 (98.0%) | 36,624 (98.1%) | 28,422 (97.8%) |  |
| Asian | 572 (0.9%) | 315 (0.8%) | 257 (0.9%) |  |
| Black | 305 (0.5%) | 161 (0.4%) | 144 (0.5%) |  |
| Mixed | 86 (0.1%) | 40 (0.1%) | 46 (0.2%) |  |
| Other | 393 (0.6%) | 212 (0.6%) | 181 (0.6%) |  |
| Unknown | 65,046 (98.0%) | 36,624 (98.1%) | 28,422 (97.8%) |  |
| Most Deprived quintile | 11,548 (16.7%) | 6,644 (17.0%) | 4,904 (16.2%) | <0.001 |
| Least Deprived quintile | 15,727 (22.7%) | 8,634 (22.2%) | 7,093 (23.4%) |  |
| Heart Failure | 12,866 (18.6%) | 7,386 (18.9%) | 5,480 (18.1%) | 0.003 |
| Hypertension | 42,714 (61.6%) | 22,559 (57.9%) | 20,155 (66.5%) | <0.001 |
| Diabetes | 10,206 (14.7%) | 6,244 (16.0%) | 3,962 (13.1%) | <0.001 |
| Stroke | 16,049 (23.2%) | 8,448 (21.7%) | 7,601 (25.1%) | <0.001 |
| Vascular disease | 12,250 (17.7%) | 8,246 (21.2%) | 4,004 (13.2%) | <0.001 |
| Antiplatelet use | 36,883 (53.2%) | 21,052 (54.0%) | 15,831 (52.2%) | <0.001 |
| CHA_2_DS_2_VASc (Mean) | 3.3 (1.8) | 2.7 (1.7) | 4.1 (1.6) | <0.001 |
| CHA_2_DS_2_VASc |  |  |  | <0.001 |
| 0 | 3,801 (5.5%) | 3,801 (9.8%) | 0 (0.0%) |  |
| 1 | 7,525 (10.9%) | 6,030 (15.5%) | 1,495 (4.9%) |  |
| 2 | 11,968 (17.3%) | 8,619 (22.1%) | 3,349 (11.0%) |  |
| 3 | 14,784 (21.3%) | 8,393 (21.5%) | 6,391 (21.1%) |  |
| 4 | 14,059 (20.3%) | 5,813 (14.9%) | 8,246 (27.2%) |  |
| 5 | 8,831 (12.7%) | 3,887 (10.0%) | 4,944 (16.3%) |  |
| 6 | 5,479 (7.9%) | 1,711 (4.4%) | 3,768 (12.4%) |  |
| 7 | 2,201 (3.2%) | 601 (1.5%) | 1,600 (5.3%) |  |
| 8 | 569 (0.8%) | 123 (0.3%) | 446 (1.5%) |  |
| 9 | 74 (0.1%) | 0 (0.0%) | 74 (0.2%) |  |

Legend: OAC – oral anticoagulants.

| **Year** | **N at risk per year** | **First event** | **Follow-up (patient-years)** | **Cumulative incidence rate (first event)** | **Incidence per 100-patient years** | **Total events** | **Cumulative incidence rate (total events)** |
| --- | --- | --- | --- | --- | --- | --- | --- |
| 1999 | 4748 | 49 | 4071.57 | 1.03 | 1.20 | 70 | 1.47 |
| 2000 | 8813 | 94 | 7582.25 | 1.07 | 1.24 | 142 | 1.61 |
| 2001 | 12504 | 156 | 10907.65 | 1.25 | 1.43 | 251 | 2.01 |
| 2002 | 15930 | 227 | 13819.72 | 1.42 | 1.64 | 381 | 2.39 |
| 2003 | 18855 | 294 | 16409.24 | 1.56 | 1.79 | 502 | 2.66 |
| 2004 | 21476 | 381 | 18590.26 | 1.77 | 2.05 | 721 | 3.36 |
| 2005 | 23513 | 403 | 20432.00 | 1.71 | 1.97 | 742 | 3.16 |
| 2006 | 25510 | 469 | 22208.86 | 1.84 | 2.11 | 860 | 3.37 |
| 2007 | 27419 | 472 | 23907.10 | 1.72 | 1.97 | 883 | 3.22 |
| 2008 | 29262 | 572 | 25548.30 | 1.95 | 2.24 | 1076 | 3.68 |
| 2009 | 31178 | 644 | 27395.42 | 2.07 | 2.35 | 1217 | 3.90 |
| 2010 | 33811 | 730 | 29755.80 | 2.16 | 2.45 | 1385 | 4.10 |
| 2011 | 36352 | 755 | 32158.03 | 2.08 | 2.35 | 1444 | 3.97 |
| 2012 | 39216 | 787 | 34814.61 | 2.01 | 2.26 | 1406 | 3.59 |
| 2013 | 41791 | 817 | 37087.68 | 1.95 | 2.20 | 1525 | 3.65 |
| 2014 | 45357 | 947 | 40847.93 | 2.09 | 2.32 | 1746 | 3.85 |
| 2015 | 52075 | 945 | 47470.88 | 1.81 | 1.99 | 1975 | 3.79 |
| **Total** |  | 8742 | 413007.32 |  | 2.12 | 16326 |  |

**Supplementary Table 3 –** Annual event and incidence rates

**Supplementary Table 4 -** Annual event rates for men and women (main analysis)

|  | **Men** | | | | **Women** | | | | **Adjusted HR for Women*** | **95%CI** |
| --- | --- | --- | --- | --- | --- | --- | --- | --- | --- | --- |
| **Year** | **Number** | **Events** | **Rate** | **95%CI** | **Number** | **Event** | **Rate** | **95%CI** |  |  |
| 1999 | 3372 | 21 | 0.92 | 0.52, 1.31 | 3114 | 28 | 1.33 | 0.83, 1,82 | 1.08 | 0.60, 1.97 |
| 2000 | 6186 | 39 | 1 | 0.68, 1.31 | 5991 | 60 | 1.42 | 1.06, 1,78 | 1.39 | 0.90, 2.14 |
| 2001 | 9206 | 81 | 1.38 | 1.08, 1.68 | 8639 | 81 | 1.31 | 1.02, 1,60 | 0.79 | 0.57, 1.10 |
| 2002 | 11812 | 85 | 1.16 | 0.91, 1.41 | 11015 | 135 | 1.84 | 1.53, 2.15 | 1.33 | 1.00, 1.76 |
| 2003 | 14239 | 134 | 1.49 | 1.23, 1.74 | 13093 | 154 | 1.79 | 1.51, 2.07 | 0.95 | 0.75, 1.22 |
| 2004 | 16513 | 175 | 1.72 | 1.46, 1.98 | 15140 | 200 | 2.04 | 1.76, 2.33 | 0.94 | 0.76, 1.16 |
| 2005 | 18524 | 177 | 1.58 | 1.34, 1.81 | 16955 | 219 | 2.03 | 1.76, 2.30 | 1.02 | 0.83, 1.26 |
| 2006 | 20553 | 184 | 1.5 | 1.28, 1.72 | 18481 | 284 | 2.45 | 2.16, 2.73 | 1.36 | 1.12, 1.65 |
| 2007 | 22566 | 180 | 1.32 | 1.12, 1.51 | 20010 | 282 | 2.21 | 1.95, 2.47 | 1.45 | 1.19, 1.77 |
| 2008 | 24573 | 253 | 1.71 | 1.50, 1.93 | 21350 | 316 | 2.36 | 2.10, 2.62 | 1.08 | 0.90, 1.28 |
| 2009 | 26355 | 277 | 1.75 | 1.54, 1.95 | 22760 | 367 | 2.57 | 2.31, 2.83 | 1.21 | 1.02, 1.43 |
| 2010 | 28458 | 321 | 1.87 | 1.66, 2.07 | 24259 | 410 | 2.7 | 2.43, 2.96 | 1.16 | 0.99, 1.35 |
| 2011 | 30238 | 308 | 1.62 | 1.44, 1.80 | 25497 | 444 | 2.73 | 2.48, 2.99 | 1.36 | 1.17, 1.59 |
| 2012 | 32233 | 351 | 1.73 | 1.55, 1.92 | 26947 | 436 | 2.5 | 2.26, 2.73 | 1.21 | 1.04, 1.40 |
| 2013 | 34315 | 377 | 1.71 | 1.54, 1.89 | 28190 | 437 | 2.41 | 2.18, 2.63 | 1.20 | 1.04, 1.38 |
| 2014 | 36033 | 462 | 1.9 | 1.73, 2.07 | 29255 | 490 | 2.5 | 2.28, 2.72 | 1.08 | 0.95, 1.24 |
| 2015 | 37994 | 429 | 1.54 | 1.40, 1.69 | 30348 | 503 | 2.24 | 2.05, 2.44 | 1.19 | 1.05, 1.36 |

* unadjusted event rates are provided for men and women. Adjustment hazard ratio for age and CHA_2_DS_2_VASc.

**Supplementary Table 5 -** Sensitivity analysis without censoring in first 3 months: annual event rates for men and women

| **Year** | **N at risk per year** | **First event** | **Follow-up (patient-years)** | **Cumulative incidence rate (first event)** | **Incidence per 100-patient years** | **Total events** | **Cumulative incidence rate (total events)** |
| --- | --- | --- | --- | --- | --- | --- | --- |
| 1999 | 6486 | 64 | 4215.23 | 0.99 | 1.52 | 94 | 1.45 |
| 2000 | 12177 | 135 | 7857.73 | 1.11 | 1.72 | 204 | 1.68 |
| 2001 | 17845 | 218 | 11315.88 | 1.22 | 1.93 | 341 | 1.91 |
| 2002 | 22827 | 296 | 14347.20 | 1.3 | 2.06 | 489 | 2.14 |
| 2003 | 27332 | 389 | 17061.27 | 1.42 | 2.28 | 655 | 2.4 |
| 2004 | 31653 | 472 | 19375.16 | 1.49 | 2.44 | 876 | 2.77 |
| 2005 | 35479 | 528 | 21351.75 | 1.49 | 2.47 | 975 | 2.75 |
| 2006 | 39034 | 615 | 23202.99 | 1.58 | 2.65 | 1113 | 2.85 |
| 2007 | 42576 | 646 | 24994.92 | 1.52 | 2.58 | 1182 | 2.78 |
| 2008 | 45923 | 784 | 26759.73 | 1.71 | 2.93 | 1489 | 3.24 |
| 2009 | 49115 | 868 | 28706.21 | 1.77 | 3.02 | 1763 | 3.59 |
| 2010 | 52717 | 994 | 31137.81 | 1.89 | 3.19 | 1878 | 2.56 |
| 2011 | 55735 | 1020 | 33576.16 | 1.83 | 3.04 | 1962 | 3.52 |
| 2012 | 59180 | 1077 | 36227.95 | 1.82 | 2.97 | 1923 | 3.25 |
| 2013 | 62505 | 1106 | 38516.78 | 1.77 | 2.87 | 2048 | 3.28 |
| 2014 | 65288 | 1221 | 42228.64 | 1.87 | 2.89 | 2258 | 3.46 |
| 2015 | 68342 | 1128 | 48610.23 | 1.65 | 2.32 | 2332 | 3.41 |
| **Total** |  | 11561 | 429485.65 |  | 2.69 | 21582 |  |

**Supplementary Table 6 –** Sensitivity analysis without censoring in first 3 months: score discrimination and reclassification

|  | **C-index** | | **IDI and Continuous NRI** | | | | | |
| --- | --- | --- | --- | --- | --- | --- | --- | --- |
| **Year** | **CHA_2_DS_2_VASC** | **CHA_2_DS_2_VA** | **IDI** | **95%CI** | **p-value** | **NRI** | **95%CI** | **p-value*** |
| 1999 | 0.68 | 0.68 | 0 | -0.002, 0.001 | 0.804 | -0.086 | -0.214, 0.194 | 0.346 |
| 2000 | 0.64 | 0.63 | 0 | -0.001, 0.001 | 0.671 | -0.085 | -0.185, 0.007 | 0.08 |
| 2001 | 0.66 | 0.67 | 0.001 | 0, 0.002 | 0.073 | 0.02 | -0.076, 0.219 | 0.638 |
| 2002 | 0.70 | 0.69 | -0.001 | -0.002, 0 | 0.173 | -0.111 | -0.169, -0.049 | <0.001 |
| 2003 | 0.66 | 0.66 | 0 | -0.001, 0.001 | 0.591 | -0.046 | -0.11, 0.005 | 0.066 |
| 2004 | 0.66 | 0.67 | 0 | -0.001, 0.001 | 0.485 | -0.039 | -0.089, 0.079 | 0.113 |
| 2005 | 0.68 | 0.68 | 0 | -0.001, 0.001 | 0.651 | -0.061 | -0.108, -0.013 | 0.013 |
| 2006 | 0.70 | 0.69 | -0.001 | -0.002, 0 | 0.047 | -0.12 | -0.161, -0.08 | <0.001 |
| 2007 | 0.69 | 0.68 | -0.001 | -0.002, 0 | 0.013 | -0.128 | -0.176, -0.082 | <0.001 |
| 2008 | 0.68 | 0.68 | 0 | -0.001, 0.001 | 0.645 | -0.082 | -0.119, -0.036 | <0.001 |
| 2009 | 0.69 | 0.69 | -0.001 | -0.001, 0 | 0.053 | -0.099 | -0.137, -0.063 | <0.001 |
| 2010 | 0.68 | 0.68 | 0 | -0.001, 0 | 0.159 | -0.091 | -0.130, -0.056 | <0.001 |
| 2011 | 0.68 | 0.67 | -0.001 | -0.002, 0 | 0 | -0.131 | -0.164, -0.099 | <0.001 |
| 2012 | 0.70 | 0.70 | 0 | -0.001, 0 | 0.186 | -0.094 | -0.128, -0.056 | <0.001 |
| 2013 | 0.69 | 0.69 | -0.001 | -0.001, 0 | 0.159 | -0.088 | -0.119, -0.055 | <0.001 |
| 2014 | 0.69 | 0.69 | 0 | -0.001, 0.001 | 0.97 | -0.074 | -0.104, -0.040 | <0.001 |
| 2015 | 0.69 | 0.69 | 0 | -0.001, 0 | 0.1 | -0.095 | -0.129, -0.064 | <0.001 |

*Note: *adjusted p-value for multiple comparisons: 2.94 × 10^-3^ (i.e. 0.05/17*).

**Annex A –** *I. Women and CHA_2_DS_2_VASc through the years in ESC and AHA/ACC/HRS AF guidelines*

1. *AHA/ACC/HRS 2014 AF Guideline*^1^ –text section cites Friberg’s 2012 BMJ paper and states *“women younger than 65 years of age and without other AF risk factors had a low risk for stroke,and it was concluded that anticoagulant treatment was not required”. The following recommendations state (with no differentiation for women vs men):*

*“For patients with nonvalvular AF with prior stroke, transient ischemic attack (TIA), or a CHA_2_DS_2_-VASc score of 2 or greater, oral anticoagulants are recommended.”* (Class of Recommendation I, Level of Evidence A to B depending on anticoagulant)

*“For patients with nonvalvular AF and a CHA_2_DS_2_-VASc score of 0, it is reasonable to omit antithrombotic therapy.”* (Class of Recommendation IIa, Level of Evidence B)

*“For patients with nonvalvular AF and a CHA_2_DS_2_-VASc score of 1, no antithrombotic therapy or treatment with an oral anticoagulant or aspirin may be considered”* Class of Recommendation IIb, Level of Evidence C

1. *AHA/ACC/HRS 2019 AF Guideline update*^2^ – The text adds that “*Female sex alone, however, does not convey increased risk in the absence of other factors*”, and the recommendations state:

*“For patients with AF and an elevated CHA_2_DS_2_ -VASc score of 2 or greater in men or 3 or greater in women, oral anticoagulants are recommended.”* Class of Recommendation I, Level of Evidence A

*“For patients with AF (except with moderate-to-severe mitral stenosis or a mechanical heart valve) and a CHA_2_DS_2_ -VASc score of 0 in men or 1 in women, it is reasonable to omit anticoagulant therapy”* Class of Recommendation IIa, Level of Evidence B

*“For patients with AF (except with moderate-to-severe mitral stenosis or a mechanical heart valve) and a CHA_2_DS_2_ -VASc score of 1 in men and 2 in women, prescribing an oral anticoagulant to reduce thromboembolic stroke risk may be considered”* Class of Recommendation IIb, Level of Evidence C-LD

1. *AHA/ACC/HRS 2023 AF Guideline*^3^ recommendation: *“For patients with AF and an estimated annual thromboembolic risk of ≥2% per year (eg,CHA_2_DS_2_-VASc score of ≥2 in men and ≥3 in women), anticoagulation is recommended to prevent stroke and systemic thromboembolism.“* (Class of Recommendation I, Level of Evidence A)

*“For patients with AF and an estimated annual thromboembolic risk of ≥1% but <2% per year (equivalent to CHA_2_DS_2_-VASc score of 1 in men and 2 in women), anticoagulation is reasonable to prevent stroke and systemic thromboembolism”* (Class of Recommendation IIa, Level of Evidence A)

No mention or specific recommendation for women as an isolated risk factor/CHA_2_DS_2_VAS score of 1 in women.

1. *ESC 2012 AF guideline update*^4^– text section: *“Female gender independently increases the risk of stroke overall unless the criterion of ‘age <65 and lone AF’ is clearly fulfilled, whereby female gender does not independently increase stroke risk.”* referencing Friberg 2012 BMJ^5^

“*Female patients with gender alone as a single risk factor (still a CHA_2_DS_2_-VASc score of 1) would not need anticoagulation if they clearly fulfil the criteria of ‘age < 65 and lone AF’”*, also highlighted in “Figure 1 – Choice of anticoagulant”, and recommendations:

*“Antithrombotic therapy to prevent thromboembolism is recommended for all patients with AF, expect in those patients (both male and female) who are at low risk (aged <65 years and lone AF), or with contraindications*” – Class of Recommendation I, Level of Evidence A”

*“Female patients who are aged <65 and have lone AF (but still have a CHA_2_DS_2_VASc score of 1 by virtue of their gender”) are low risk and no antithrombotic should be considered*” – Class of Recommendation IIa, Level of Evidence B”

1. *ESC 2016 AF Guideline*^6^ – text section: *“an individualized weighing of risk, as well as patient preferences, should inform the decision to anticoagulate patients with only one CHA_2_DS_2_-VASc risk factor, apart from female sex. Female sex does not appear to increase stroke risk in the absence of other stroke risk factors”*

No mention or specific recommendation for women as an isolated risk factor/CHA_2_DS_2_VAS score of 1 in women.

1. *ESC 2020 AF guideline*^7^ – text section: *“Female sex is an age-dependent stroke risk modifier rather than a risk factor per se. Observational studies showed that women with no other risk factors (CHA_2_DS_2_-VASc score of 1) have a low stroke risk, similar to men with a CHA_2_DS_2_-VASc score of 0”*, referencing Friberg 2012 BMJ study.

*“The simplified CHA_2_DS_2_-VA score could guide the initial decision about OAC in AF patients, but not considering the sex component would underestimate stroke risk in women with AF.”*

Recommendation: “*OAC is recommended for stroke prevention in AF patients with CHA_2_DS_2_-VASc score ≥2 in men or≥3 in women.”* (Class of Recommendation I, Level of Evidence A)

*“OAC should be considered for stroke prevention in AF patients with a CHA2DS2-VASc score of 1 in men or 2 in women”* (Class of Recommendation IIa, Level of Evidence B)

No specific recommendation for women as an isolated risk factor/CHA_2_DS_2_VAS score of 1 in women.

*II. Potential simplification of current recommendations (2020 ESC & 2023 AHA/ACC/HRS) utilizing CHA_2_DS_2_VA:*

- OAC is recommended for stroke prevention in AF patients with CHA_2_DS_2_-VA score ≥2
- For patients with AF and an estimated annual thromboembolic risk of ≥2% per year (eg,CHA_2_DS_2_-VA score of ≥2), anticoagulation is recommended to prevent stroke and systemic thromboembolism.
- For patients with AF and an estimated annual thromboembolic risk of ≥1% but <2% per year (equivalent to CHA_2_DS_2_-VA score of 1), anticoagulation is reasonable to prevent stroke and systemic thromboembolism

*III.* *Asian and Pacific Guidelines*

1. The *Australian 2018 AF guideline*^8^ recommends the use of CHA_2_DS_2_-VA:

Text: *“The female sex category component of the score (Sc) adds the most predictive value in the presence of multiple additional risk factors. Female sex alone or in the presence of one additional risk factor does not confer sufficient or consistent increased risk”*

*“Most guidelines have adopted the cumbersome practice of selecting different CHA_2_DS_2_-VASc thresholds for males and females when recommending anticoagulation. To avoid this practice, we recommend a sexless CHA_2_DS_2_-VASc score (i.e. removing female sex), abbreviated as CHA_2_DS_2_-VA score in these guidelines, and we provide one consistent recommendation for both sexes”*

Recommendations: *“Oral anticoagulation therapy to prevent stroke and systemic embolism is recommended in patients with N-VAF whose CHA_2_DS_2_-VA score is 2 or more, unless there are contraindications to anticoagulation”.* (GRADE quality of evidence: High; GRADE strength of recommendation: Strong.)

*“Oral anticoagulation therapy to prevent stroke and systemic embolism should be considered in patients with N-VAF whose CHA_2_DS_2_-VA score is 1.”* (GRADE quality of evidence: Moderate; GRADE strength of recommendation: Strong.)

*“Oral anticoagulation therapy to prevent thromboembolism and systemic embolism is not recommended in patients with N-VAF whose CHA_2_DS_2_-VA score is 0.”* (GRADE quality of evidence: Moderate; GRADE strength of recommendation: Weak.)

1. *2021* *Focused update Consensus Guideline APHRS stroke prevention in AF* ^9^

*“Figure 8 – Three-step approach for the use of OACs for stroke prevention in AF.”*

Recommendations: *“For stroke risk assessment, a risk-factor-based approach is recommended, using the CHA_2_DS_2_-VASc stroke risk score to initially identify patients at “low stroke risk” (CHA_2_DS_2_-VASc = 0 in men, or 1 in women) who should not be offered antithrombotic therapy.”*

*“In AF patients with CHA_2_DS_2_-VASc score ≥2 in men or ≥3 in women, OAC is recommended for stroke prevention”*

*“In AF patients with a CHA_2_DS_2_-VASc score of 1 in men or 2 in women, OAC should be considered for stroke prevention.”*

**References**

1.January CT, Wann LS, Alpert JS, Calkins H, Cigarroa JE, Cleveland JC Jr, Conti JB, Ellinor PT, Ezekowitz MD, Field ME, Murray KT, Sacco RL, Stevenson WG, Tchou PJ, Tracy CM, Yancy CW; American College of Cardiology/American Heart Association Task Force on Practice Guidelines. 2014 AHA/ACC/HRS guideline for the management of patients with atrial fibrillation: a report of the American College of Cardiology/American Heart Association Task Force on Practice Guidelines and the Heart Rhythm Society. J Am Coll Cardiol. 2014;64:e1-76. doi: 10.1016/j.jacc.2014.03.022.

2. January CT, Wann LS, Calkins H, Chen LY, Cigarroa JE, Cleveland JC Jr, Ellinor PT, Ezekowitz MD, Field ME, Furie KL, Heidenreich PA, Murray KT, Shea JB, Tracy CM, Yancy CW. 2019 AHA/ACC/HRS Focused Update of the 2014 AHA/ACC/HRS Guideline for the Management of Patients With Atrial Fibrillation: A Report of the American College of Cardiology/American Heart Association Task Force on Clinical Practice Guidelines and the Heart Rhythm Society. J Am Coll Cardiol. 2019;74:104-132. doi: 10.1016/j.jacc.2019.01.011.

3. Writing Committee Members; Joglar JA, Chung MK, Armbruster AL, Benjamin EJ, Chyou JY, Cronin EM, Deswal A, Eckhardt LL, Goldberger ZD, Gopinathannair R, Gorenek B, Hess PL, Hlatky M, Hogan G, Ibeh C, Indik JH, Kido K, Kusumoto F, Link MS, Linta KT, Marcus GM, McCarthy PM, Patel N, Patton KK, Perez MV, Piccini JP, Russo AM, Sanders P, Streur MM, Thomas KL, Times S, Tisdale JE, Valente AM, Van Wagoner DR. 2023 ACC/AHA/ACCP/HRS Guideline for the Diagnosis and Management of Atrial Fibrillation: A Report of the American College of Cardiology/American Heart Association Joint Committee on Clinical Practice Guidelines.

J Am Coll Cardiol. 2024 Jan 2;83(1):109-279. doi: 10.1016/j.jacc.2023.08.017.

4. Camm AJ, Lip GY, De Caterina R, Savelieva I, Atar D, Hohnloser SH, Hindricks G, Kirchhof P; ESC Committee for Practice Guidelines (CPG). 2012 focused update of the ESC Guidelines for the management of atrial fibrillation: an update of the 2010 ESC Guidelines for the management of atrial fibrillation. Developed with the special contribution of the European Heart Rhythm Association. Eur Heart J. 2012;33:2719-47. doi: 10.1093/eurheartj/ehs253.

5. Friberg L, Benson L, Rosenqvist M, Lip GY. Assessment of female sex as a risk factor in atrial fibrillation in Sweden: nationwide retrospective cohort study. BMJ. 2012;344:e3522. doi: 10.1136/bmj.e3522.

6. Kirchhof P, Benussi S, Kotecha D, Ahlsson A, Atar D, Casadei B, Castella M, Diener HC, Heidbuchel H, Hendriks J, Hindricks G, Manolis AS, Oldgren J, Popescu BA, Schotten U, Van Putte B, Vardas P; ESC Scientific Document Group. 2016 ESC Guidelines for the management of atrial fibrillation developed in collaboration with EACTS. Eur Heart J. 2016 Oct 7;37(38):2893-2962. doi: 10.1093/eurheartj/ehw210.

7. Hindricks G, Potpara T, Dagres N, Arbelo E, Bax JJ, Blomström-Lundqvist C, Boriani G, Castella M, Dan GA, Dilaveris PE, Fauchier L, Filippatos G, Kalman JM, La Meir M, Lane DA, Lebeau JP, Lettino M, Lip GYH, Pinto FJ, Thomas GN, Valgimigli M, Van Gelder IC, Van Putte BP, Watkins CL; ESC Scientific Document Group. 2020 ESC Guidelines for the diagnosis and management of atrial fibrillation developed in collaboration with the European Association for Cardio-Thoracic Surgery (EACTS): The Task Force for the diagnosis and management of atrial fibrillation of the European Society of Cardiology (ESC) Developed with the special contribution of the European Heart Rhythm Association (EHRA) of the ESC. Eur Heart J. 2021;42:373-498. doi: 10.1093/eurheartj/ehaa612.

8. NHFA CSANZ Atrial Fibrillation Guideline Working Group. National Heart Foundation of Australia and the Cardiac Society of Australia and New Zealand: Australian clinical guidelines for the diagnosis and management of atrial fibrillation 2018. Heart Lung Circ. 2018; 27: 1209-1266.

9. Chao T-F, Joung B, Takahashi Y, Lim TW, Choi FK, Chan YH, Guo Y, Sriratanasathavorn C, Oh S, Okumura K, Lip GYH.. 2021 Focused Update Consensus Guidelines of the Asia Pacific Heart Rhythm Society on Stroke Prevention in Atrial Fibrillation: Executive Summary. Thromb Haemost. 2022;122:20–47.
